# Computed tomography-guided biopsies in children: accuracy, efficiency and dose usage

**DOI:** 10.1186/s13052-016-0319-7

**Published:** 2017-01-06

**Authors:** Tatjana Gruber-Rouh, Axel Thalhammer, Thomas Klingebiel, Nour-Eldin A. Nour-Eldin, Thomas. J. Vogl, Katrin Eichler, Nagy Naguib, Martin Beeres

**Affiliations:** 1 Institute for Diagnostic and Interventional Radiology, University Hospital, Theodor-Stern-Kai 7, 60590 Frankfurt am Main, Germany; 2 Clinic for Pediatric and Adolescent Medicine, University Hospital, Theodor-Stern-Kai 7, 60590 Frankfurt am Main, Germany; 3 Department of Diagnostic and Interventional Radiology, Cairo University Hospital, Cairo, Egypt; 4 Department of Diagnostic and Interventional Radiology, Alexandria University Hospital, Alexandria, Egypt

**Keywords:** Biopsy, Children, CT-guidance

## Abstract

**Background:**

Computed-tomography-guided interventions are attractive for tissue sampling of paediatric tumor lesions; however, it comes with exposure to ionizing radiation. The aim of this study was to analyse the radiation dose, accuracy and speed of CT-guided interventions in paediatric patient cohort.

**Methods:**

We retrospectively reviewed CT-guided interventions over a 10 -year period in 65 children. The intervention site consisted of bones in 38, chest (lung) in 15 and abdomen (liver, lymph nodes) in 12 cases. Radiation dose and duration of the procedures were analysed. The statistical analysis was performed using dedicated statistical software (BiAS 8.3.6 software, Epsilon Verlag, North Hasted).

**Results:**

All interventions were performed successfully. Mean target access path to lesion within the patients was 6.0 cm (min 3.5 cm, max 11.2 cm). Time duration to complete intervention was 25:15 min (min 17:03 min, max 43:00 min). The dose-length product (DLP) of intervention scan was 29.5 mGy · cm (min 6 mGy · cm, max 85 mGy · cm) with the lowest dose for biopsies in the region of the chest (*p* = 0.04).

**Conclusions:**

With justified indications, CT-guided paediatric interventions are safe, effective and can be performed both, with short intervention times and low radiation exposure.

## Background

Image-guided biopsies are increasingly used to establish a diagnosis of benign and malignant lesions in adults. For radiation safety purpose, clearly justified indication for CT-guided intervention in children is necessary and sonography or magnetic resonance imaging (MRI) are to be preferred whenever possible [1].

Compared to other imaging methods for biopsy, such as MRI or ultrasound, CT offers an accurate image quality with a high resolution. CT is a safe method to perform biopsies of pulmonary and bony lesions, especially if they are in a complex localisation (e.g. central lesions). As already analysed in adults, compared to open surgical biopsy, image-guided biopsies in general are faster and less invasive, so image guided biopsies provide an excellent alternative to surgery in selected cases [2–4]. Diagnostic CT-interventions can also be used in children [5]. The introduction of latest generation CT systems show that paediatric-specific CT imaging is possible adding newly introduced technology to reduce radiation exposure [6]. With justified indications and precise performance, CT-guided interventions can be successful performed in paediatric patients with limited risks [1, 5].

This retrospective study was conducted to evaluate radiation dose, accuracy and speed of CT-guided interventions in pediatric patient cohort.

## Methods

### Patient population

The study was conducted under an approval by the Institutional Review Board. The inclusion criterion for the CT-guided therapy consisted of a therapeutic relevant findings, which had been discussed and considered to be non-successful by sonographic or MRI means. All intervention procedures were performed after informed consent of the parents and patient (≤18 years) had been obtained and in the absence of contraindications for CT-guided interventions. A special consent for evaluation of the dataset was waived by the Institutional Review board as the data were evaluated retrospectively.

Enclosed in this retrospective single-centre study have been 65 children. We report our early experience with the technique and assess its place and efficacy in the clinical management. Sixty-five children (25 female, 40 male) were punctured with CT-guiding. At the time of the intervention, the mean age of the children was 12.3 years (age range, 1–18 years). The needle intervention of bones, chest (lung) and abdomen (liver, lymph nodes) was performed in 38, 15 and in 12 patients respectively. In every patient a diagnostic puncture was performed.

The CT-guided interventions were carried out by three radiologists with each more than 10 years of experience in CT-guided interventions.

### Inclusion criteria

All patients were previously evaluated in the multidisciplinary conference (paediatrics, paediatric oncologist, pathologist, surgery, radiation therapy and radiologist) and selected for CT-guided intervention.

The CT-guided intervention was indicated for targets that were considered suspicious based on clinical and morphological results after CT, MRI or PET-CT examinations. The coagulation profile of each patient was checked before the procedure and did not extend given values: INR < 1.5; Platelet count > 25.000 (μL); according to up-to-date interventional radiology guidelines.

### Exclusion criteria

Also patients older than 18 years were excluded for this retrospective analysis.

Patients with targets that were accessible by ultrasound or MRI did not receive a CT-guided intervention because of radiation safety purpose.

Combined procedures (CT-Intervention plus ablation and/or surgery) were excluded because the rate of complications might not be comparable to our tissue-sampling cohort.

### CT-guided intervention

Lung biopsies and interventions of children before the age of 15 required general anaesthesia. The remaining interventions were performed with local anaesthesia.

A 16-slice CT scanner (Siemens Somatom Sensation, Siemens Healthcare, Erlangen, Germany) and a 64-slice CT scanner (Somatom Definition AS, Siemens Healthcare, Erlangen, Germany) were used for interventions. The CT scan could be reduced to the minimum scan-range of the suspected tissue, since diagnostic imaging modalities (Ultrasound, MRI or CT) have proven the suspected tissue or lesion before intervention. There was no need to perform an additional full-coverage CT scan of the suspected region.

First, a helical CT-scan limited to the suspected region of the body was acquired; the dataset was transferred to a 3D planning workstation (SyngoVia, Siemens, Erlangen, Germany) to plan the needle path. Following, the CT-table was moved to the planned position. The laser grid of the gantry, illuminating the plane in the centre of the gantry, was activated. A marker grid was placed at the estimated position of the entry point on the skin of the patient. The skin entry point was marked with a felt-tip pen. Before introduction of a biopsy needle, local anaesthesia was applied.

After onset of the local anesthesia, sterile draping following a small skin incision at the needle entry point was performed. By acquiring a single slice image, the position of the needle could be checked.

A coaxial approach was used for CT-guided intervention of soft parts. This technique is characterized by a combination of two needles: a puncture sheath (Puncture Sheath, Somatex® Medical Technologies GmbH, Germany) and biopsy-handy (Biopsy Handy, Somatex® Medical Technologies GmbH, Germany).

The sheath - a thicker, shorter needle is inserted down to the anterior edge of the lesion. Then, the biopsy-handy - a thinner, longer needle is introduced through the sheath. Finally, the biopsy needle is inserted into the edge of the lesion for tissue sampling. Multiple samples can be taken using the thinner biopsy needle without several skin-punctures due to the use of the sheath in the coaxial approach.

Because of the risk for a pneumothorax, biopsies of lung were performed with a biopsy needle without puncture sheath. The CT-guided intervention of bone was performed with a special bone biopsy needle (SAFE-CUT Biopsy System, Somatex® Medical Technologies GmbH, Germany).

The biopsy needle was removed after adequate specimens were taken. Since no on-site cytopath was available, we had to rely on our acquired CT scan that the specimen was taken out successful first step. The specimen collection was sent to the pathology department according to the intervention.

Upon completing the entire procedure, a control scan of the target region including all adjacent structures should follow in order to rule out immediate complications. After the intervention a sterile patch was used to close the skin-entry.

### Data evaluation

#### Quantitative and statistical evaluation

All patient data were evaluated retrospectively. Clinical characteristics, including procedural details, success and complications, were determined. The evaluation of the clinical data was performed by analysing the medical records of the patients. Data were reported at event occurrence. The analysis of the CT studies as well as the intervention-data was performed by two radiologists.

### Clinical outcome

Evaluation criteria included technical success as well as major and minor complications according to the “Society of Interventional Radiology” guidelines [7]. CT-guided intervention was considered technical successful if the region of interest was reached, specimen taken and pathological findings were corresponding, analogous SIR-Guidelines [8].

Major complications included any undesirable events occurring as results of intervention were recorded. Minor complications were those that required no management, except appropriate monitoring and intense follow-up analogous SIR-Guidelines.

### Radiologic outcome

CT-guided interventions were compared according to the following parameters of intervention: radiation dose, procedure duration, number of needle correction scans and skin-to-lesion length.

Dose-length products (DLP) of CT and intervention scans were documented in the patient protocol of the CT scan.

In order to compare the exposure to radiation, the number of images taken during each intervention was recorded. Time of the procedure “beginning of intervention to the end of the intervention” was performed by using the CT-intervention planning scan as beginning, and the last image acquired as the end, which in our cohort was the control scan to rule out complications.

The statistical analysis was performed using BiAS 8.3.6 software (Epsilon Verlag, North Hasted). Statistical significance was calculated according to the Mann-Whitney-*U*-test.

## Results

All CT interventions (*n* = 65) were performed without any serious side effects. Mean target access path within the patients was 6.0 cm (min 3.5 cm, max 11.2 cm). The mean target access path within the bone-group was 4.2 cm (min 4.0 cm, max 11.2 cm), 3.8 cm (min 3.7 cm, max 7.9 cm) in the chest-group and 4.9 cm (min 4.7 cm, max 9.2 cm) in the abdomen-group. The analyses showed no statistical differences among the groups (*ρ* = 0.15).

The duration to complete the intervention was 25:15 min (min 17:03 min, max 43:00 min). The duration to obtain the correct puncture site on skin was 8:06 min while the average “skin-to-target” time was 3:20 min. The mean duration of “target achieved to intervention end” was 04:23 min.

**Fig. 1:**
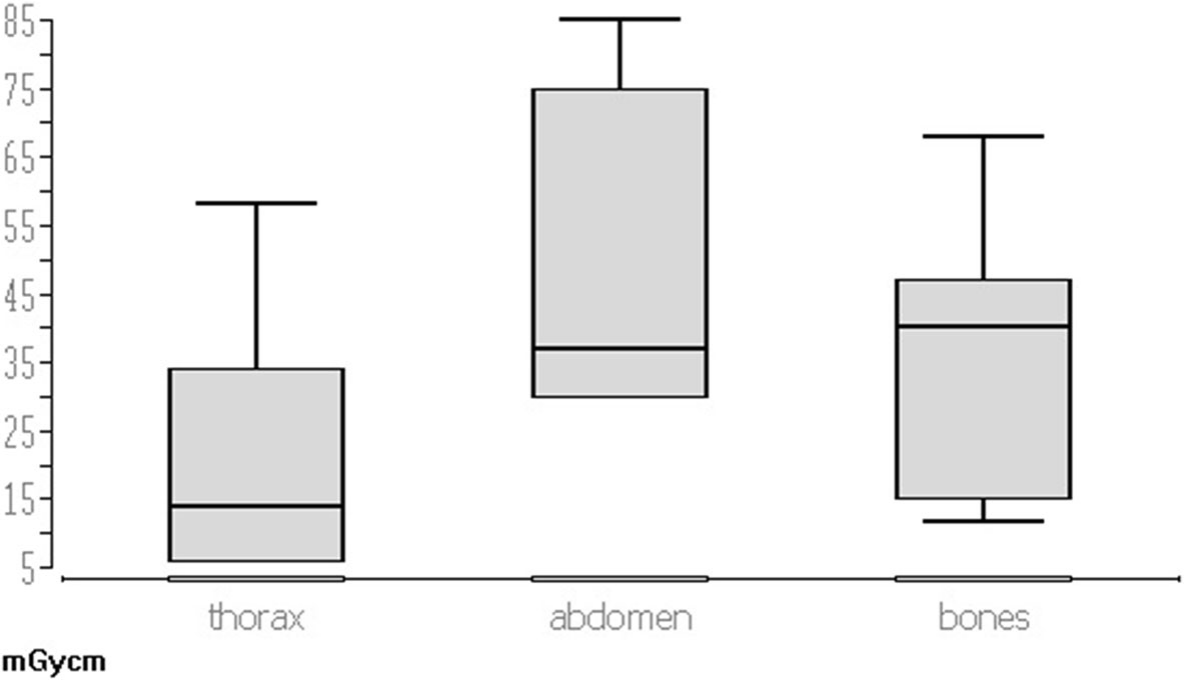
The box-plot shows dose-length products (DLP) of intervention scans in regard to region of intervention

The dose-length product (DLP) of the intervention scan was 29.5 mGy · cm (min 6 mGy · cm, max 85 mGy · cm) with the lowest dose for biopsies in the region of the chest. The DLP of the intervention scan in the chest was 14.8 mGy · cm. The DLP of intervention scan in the abdomen was 35.0 mGy · cm and 38.4 mGy · cm in the bone with the lowest value for biopsies of the extremities (15 mGy · cm). The results were statistically significant in according to the Mann-Whitney-*U* test (*p* = 0.04) (Fig. 1).

The average number of images needed for needle correction was 3.3 (min 3, max 10).

### Clinical outcome

CT-guided intervention was successful all of our procedures (100%). No major complications occurred. Concerning minor complications, a pneumothorax (1.5%) and a focal haemorrhage (4%) with haemoptysis (1.5%) were observed after intervention of the lung and 2 cases of focal haemorrhage following intervention of osseous lesions (3%), which were clinically not relevant. There was no need for additional surgical treatments or interventions in our cohort.

**Fig. 2:**
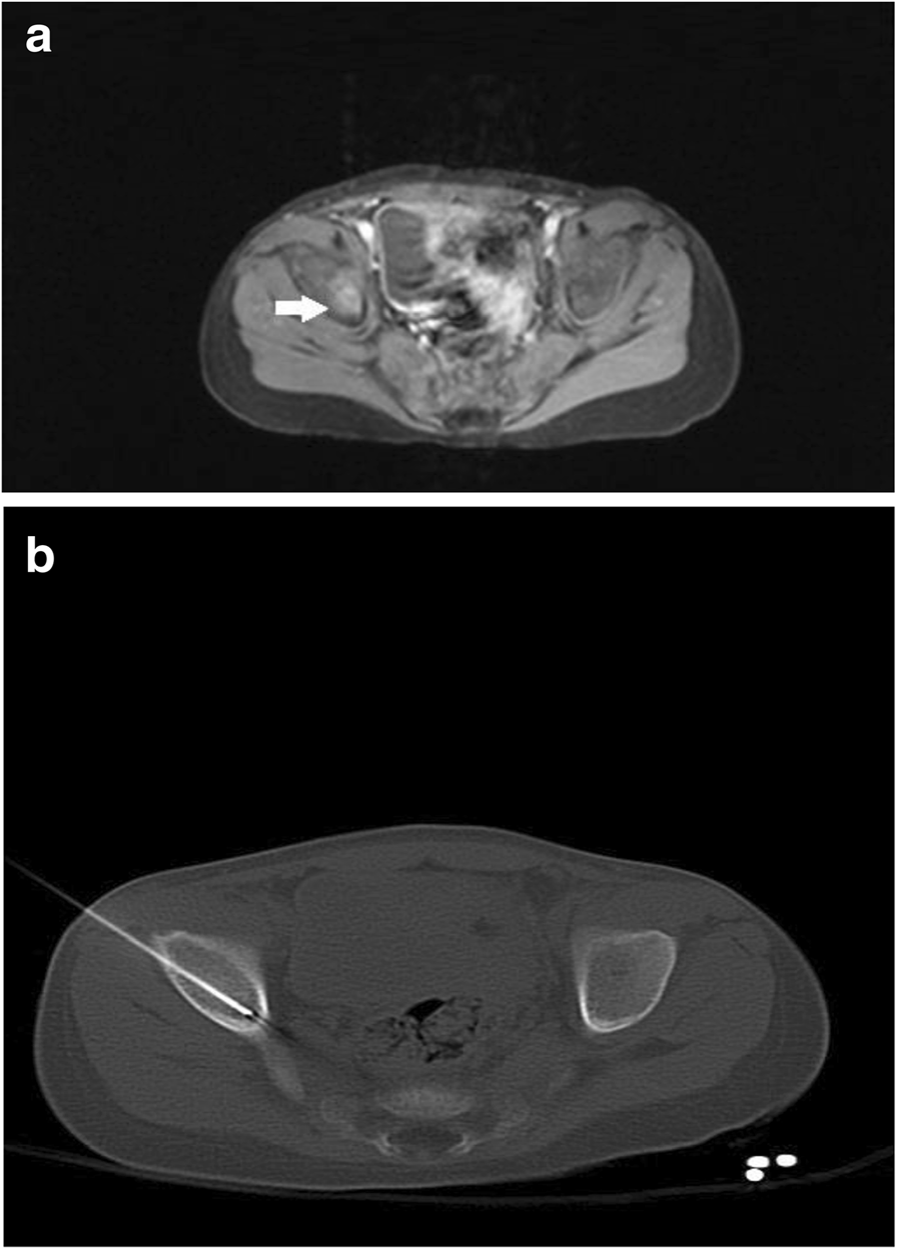
Five-year-old child with neurooblastoma. In April 2012 the patient had in follow up MRI (a) a suspect lesion with a size of 11 × 14 mm in os ileum right. In MRI no further metastatic lesions were documented. The intervention of os ileum was performed with CT guidance. The CT-guided intervention was performed without complications. The pathological report revealed a metastasis from neuroblastoma. **a**. Enhanced transverse T1-weighted MR image with fat saturation shows a 11 × 14 mm suspect lesion in os ileum right (*arrows*). **b**. CT-Image documented a location of biopsy needle in the lesion of os ileum

**Fig. 3:**
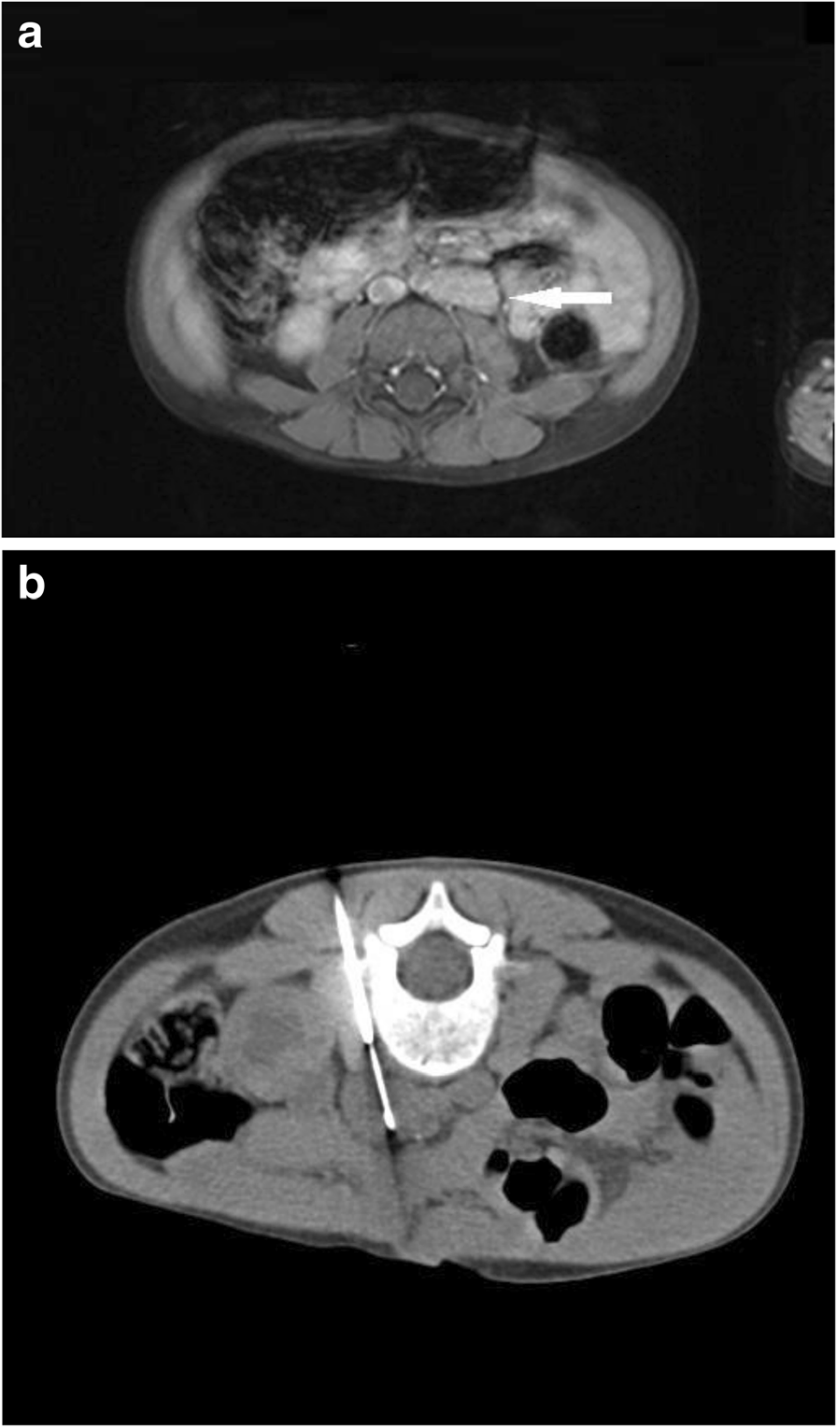
Four years old child with recurrent urinary tract infections. In MRI an unknown solid retroperitoneal masses on the left was documented. The size of the masses was 15 × 26 mm. Because of proximity the lesion to aorta and because of better controllability the intervention was performed with CT guidance. No complications were documented. The pathological report revealed a ganglioneuroma. **a**. Enhanced transverse T1-weighted MR image with fat saturation shows a suspect lesion paraaortal on the left (*arrows*). **b**. CT-Image documented a location of biopsy needle in the lesion

Malignant lesions including primary tumours and metastases were found in 55 patients (84.6%) (Fig. 2). Benign inflammatory lesions including tuberculosis were found in ten patients (15.4%) (Fig. 3).

## Discussion

Nowadays, percutaneous interventions of chest, abdomen, pelvis and bones are mostly performed by the use of image-guided techniques like ultrasound (US), magnetic resonance imaging (MRI), and computed tomography (CT) [9–14]. In general, US- and MRI- guidance is preferred whenever possible because, unlike CT-guided interventions, they are not associated with radiation exposure [15].

CT-technique offers the clinical radiologist a powerful tool to gain excellent image quality especially in complex targeted areas. The potential of various reconstructions in different axes as well as a wide-spread availability enable CT-guidance to achieve an exact needle positioning. Altogether resulting in fewer complications and higher diagnostic accuracy [15, 16]. However, the main disadvantage of CT-guided intervention remains the radiation exposure of the patient [17, 18]. The radiation dose aspect is fundamental in a pediatric patient cohort. Concerns about radiation have to be discussed seriously because long-term effects can become apparent years or decades after the examination. Therefore, minimizing radiation dose in CT is crucial as children are more sensitive to X-rays than adults [15, 19]. CT scans need to be dose-adapted, proper tube voltage and tube current reduction must be ensured using automated tube current modulation and maybe automated tube potential. For male patients gonad shields are recommended [1]. Modern CT machines offer various technical features for low-dose CT acquisition. For example the use of high-pitch dual-source CT, automated tube current and voltage selection, iterative reconstruction algorithms as well as latest generation tube-detector systems even in up-to-date single-source CT machines are able to reduce the amount of radiation exposure considerably [6, 20, 21]. We recommend using these tools whenever possible. In addition to reduce the radiation dose, post-interventional CT controls should not be performed routinely [22].

The main objective of this study was to analyse the radiation dose and speed of CT-guided interventions in children. CT-guided intervention was successful in 100% of all cases. Dose-length product (DLP) of the intervention scan was 29.5 mGy · cm (min 6 mGy · cm, max 85 mGy · cm) with the lowest dose for biopsies in the region of the chest reaching statistically significance (*p* = 0.04). Mean target access path within the patients was 6.0 cm (min 3.5 cm, max 11.2 cm). Analysis of the access path showed no statistical differences among the groups (*ρ* = 0.15). Time duration to complete intervention was 25:15 min (min 17:03 min, max 43:00 min).

One important issue referring the radiation dose and speed of CT-guided intervention is the experience of the interventional radiologist. In our study, we were able to have success in 100% of our cases.

In comparison to Shin et al, there was demonstrated a 76% success rate for biopsies in musculoskeletal lesions. Never the less, even with this success rate the procedure is accurate and safe [23].

One recent study analysed mixed Ultrasound- and CT- core needle biopsies in pediatric soft-tissue masses [24]. In this study of 84 children only 3% of the reported biopsies were non-diagnostic. However, biopsies from soft-tissue masses arising from bones as well as random biopsies of liver and kidney were excluded in this study. No information was given about the duration of the procedure as well as radiation dose when CT was used.

Actually, there is a lack of study data to compare our dose-length product (DLP) with matching reference values.

Comparing our DLP values (mean 29.5 mGy · cm) to the computer tomographic dose index (CTDIvol), recommended levels of 25 Gy for paediatric abdominal CT are accepted [24]. This means that our levels for intervention nearly matches in mean the values for a standard CT scan.

But even if the radiation dose values are low, there is always an individual risk for cancer over a patient’s lifetime due to radiation. This is proportional to the amount of radiation dose absorbed [25]. The main principle in radiation exposure is “as low as reasonably achievable” (ALARA) to reduce the incidence of radiation related cancers [24].

The duration to complete the intervention is an important fact, too. As mentioned prior the experience of the radiologist is important, on the one hand to minimize risks, on the other hand to reduce the duration to a minimum required. Comparing our mean time of duration with the Laser-Navigation-Study (LNS) in adults, our duration was a bit longer (25:15 min vs. 20:25 min) [17]. But regarding the fact, that the intervention in children is often more difficult because of the body anatomy combined with our low DLP, the elongation of the procedure might be tolerable.

Our study has several limitations worth mentioning. First, the study design is retrospective. Second, the patient population which received varying CT-guided interventions was very heterogeneous. Third, the intervention locations were different in several cases.

Prospective studies are necessary to further refine the role of CT-guided interventions in children. However, given the small number of children who require CT-guided intervention because of radiation exposure, it may be difficult to perform a prospective study with a reliable outcome.

Another important point is the use either of Gray (Gy = as the absorbed energy per unit of mass) or to compare the effective doses in Sievert, which is influenced by different tissue factors in CT [25]. However, we decided to analyse the plain given data to avoid disturbances using different tissue weighting-factors in Sievert.

## Conclusion

CT guided interventions in children, considering narrow indications, are a safe and efficient method to achieve diagnostic tissue samples in selected cases. Every case should be discussed on an individual base and evaluated for the possibility of using Ultrasound or MRI guided techniques instead of CT. If the CT guided intervention is the most effective option, it can be performed with short intervention time and low radiation exposure for pediatric patients.

## Abbreviations

CT: Computed tomography
CTDI: Computer tomographic dose index
DLP: Dose-length product
INR: International normalized ratio
LNS: Laser-Navigation-Study
MRI: Magnetic resonance imaging
PET-CT: Positron emission tomography–computed tomography
SIR-Guidelines: Society of Interventional Radiology - Guidelines
US: Ultrasound
